# Special Issue “Clinical Advances in Chronic Intestinal Diseases Treatment”

**DOI:** 10.3390/jcm11051258

**Published:** 2022-02-25

**Authors:** Eva Latorre, Jose Emilio Mesonero

**Affiliations:** 1 Instituto de Investigacion Sanitaria de Aragon (IIS Aragon), 50009 Zaragoza, Spain; mesonero@unizar.es; 2 Instituto Agroalimentario de Aragon—IA2—(Universidad de Zaragoza—CITA), 50013 Zaragoza, Spain; 3 Departamento de Bioquimica y Biologia Molecular y Celular, Facultad de Ciencias, Universidad de Zaragoza, 50009 Zaragoza, Spain; 4 Departamento de Farmacologia, Fisiologia y Medicina Legal y Forense, Facultad de Veterinaria, Universidad de Zaragoza, 50009 Zaragoza, Spain

During the last decades, the management of patients with chronic intestinal diseases has experienced remarkable progress from both diagnostic and therapeutic point of view. Irritable bowel syndrome (IBS) and inflammatory bowel disease (IBD) are the best known and with the highest incidence and prevalence around the world among chronic intestinal pathologies. Both chronic conditions display a significant overlap in terms of symptoms, pathophysiology, and treatments. However, these chronic intestinal diseases are poorly characterized and the understanding about is limited. New clinical approaches with novel mechanisms of action may offer more efficient options for treatment of chronic intestinal diseases, especially in those patients who are not optimally characterized or controlled.

Integration of innovative approaches into clinical practice together with emerging strategies for management of chronic intestinal diseases would permit the amelioration of patient outcomes, and potentially slow the progressive course of these diseases. In this Special Issue, we have collected the latest approaches to improve the management of chronic intestinal diseases. Six articles have been published in total, including both reviews and research articles.

Most of the studies are focused on IBD, a chronic inflammatory disease of the gut with heterogeneous manifestations, and a clinical presentation as Crohn’s disease (CD), ulcerative colitis (UC), or IBD unclassified (IBD-U) [1]. The prolonged inflammation of the gastrointestinal tract results in critical damage, leads to a wide range of signs and symptoms such as diarrhoea, abscesses, fistulas, abdominal pain, or stenosis, that have a significant effect on the quality of life of affected patients. The prevalence and incidence of IBD in the world is increasing, especially in developed countries. Over 2.5 million residents in Europe are estimated to have IBD, with substantial costs for health care: around €4.6–5.6 billion a year.

On the other hand, IBS is a functional gastrointestinal disorder, whose main symptoms are recurrent abdominal pain, changes in the frequency or characteristics of stool and abdominal distension, but without morphological, metabolic, or neurologic alterations. It is diagnosed using Rome IV clinical parameters and classified in 4 different subtypes according to patient’s bowel habit: IBS-D with predominant diarrhoea, IBS-C with predominant constipation, IBS-M, with alternation between diarrhoea and constipation, and IBS-U, unclassified, including individuals who do not fall into the other categories [2]. Curiously, patients with IBS-like symptoms are the single largest group of patients presenting gastrointestinal (GI) complaints in both primary and secondary healthcare. IBS has been estimated to affect at least 7–21% of the global adult population [3]. 

The exact cause of IBD and IBS are unknown. However, IBD is the result of a defective immune system. Nowadays, IBD treatment is based on biologic therapy (monoclonal antibodies against certain proteins causing inflammation). Managing IBS has attracted major attention because single-agent therapy rarely relieves bothersome symptoms for all patients. In clinical practice, there is still a lack of effective treatment for IBS, and the prescribed drugs usually alleviate only one symptom of the whole syndrome. The high incidence and prevalence of these pathologies, expensive treatments, and the increasing number of refractory patients, are sufficient reasons to seek to improve treatments based on the available drugs and develop novel clinical managements.

Biologic therapies in IBD have increased hugely in past decades; multiplying the options to treat patients, but also adding more difficulties in choosing the right treatment. Laredo et al. summarize the current data comparing biologic therapies in both, Crohn’s disease and ulcerative colitis in diverse clinical situations and synthesize the evidence related to predictors of biologic response [4]. Evidence from meta-analysis and real-world experience are valuable, but individual characteristics such as age, patient preferences, and comorbidities, as well as costs, must be contemplated to select the best treatment for the IBD patient. Despite the important benefits that biological agents bring to IBD management, in some patients the biologic therapy is ineffective; then the combination of two biological therapies seems a reasonable alternative. Brunet and Calvet offer a comprehensive update on dual biologic therapy in aggressive IBD [5]. Indeed, ustekinumab plus vedolizumab and vedolizumab plus anti-TNF were the most used co-treatments for Crohn’s disease. For ulcerative colitis, the most used co-treatments were vedolizumab plus anti-TNF and vedolizumab plus tofacitinib. These dual biologic therapies have shown good efficacy and few adverse events have been reported.

The development of biological agents was a crucial revolution for IBD management. However, despite the continuous and key advances in this area, there are still important questions to be clarified. The impact of these agents on postoperative infectious complications is uncertain, especially for the common ustekinumab and vedolizumab. García et al. has evaluated the safety of preoperative anti-TNF, vedolizumab or ustekinumab treatments in IBD patients and demonstrate that preoperative administration of biologics does not seem to be a risk factor for overall postoperative complications, although it could be for postoperative infections [6]. Beyond current biologic therapies, novel selective blockade of pro-inflammatory factors as JAK, S1P or IL-6 are other emerging strategies for IBD treatment. Kofla-Dłubacz, et al. review the latest investigations on immune selective forms of therapy in IBD, from the inhibition of the TNFα pathways, until group 12/23 cytokines, as well as lymphocyte migration [7].

On the other hand, IBS is a common functional digestive condition, where gut-brain axis is involved. Between all symptoms and alterations, IBS patients present some neurotransmitter dysfunctions that could cause disruption of gut-brain axis and would explain the onset of some IBS symptoms. Gros et al. have assessed the neurotransmitter dysfunctions in IBS and explored the potential therapeutic approaches [8]. The role of the gut-brain axis in the pathogenesis of this syndrome should be clarify for a improved management of the IBS patients

Apart from IBD and IBS, other chronic intestinal diseases need to be studied for a better understanding. Chronic intestinal pseudo-obstruction is a scarce condition with symptoms of recurrent intestinal obstruction, but without any lesions, especially relevant on infants. Appropriate management with a multidisciplinary approach and nutritional support could improve the mortality rates. In an important retrospective study, Ko, et al. have analysed the clinical outcomes and predictors of this chronic intestinal disease [9]. 

In conclusion, the treatment of IBD is evolving rapidly, while the number of biological therapies available is increasing. Despite this, and although there are numerous studies that evaluate the efficacy and safety of each therapy individually, there is a lack of direct trials that help the clinician to choose the best possible treatment. Undoubtedly, there are current recommendations to be able to select these treatments, but we must always be attentive to new drugs with good results, especially for those refractory and critical patients, while waiting for the new molecules that will be available in the future. In addition, and although IBD cannot be considered an autoimmune disease, it is true that the immune system is altered, so investigations about the immunological mechanisms involved to achieve highly selective forms of therapy with fewer side effects are needed. Similarly, other chronic intestinal diseases as IBS, intestinal pseudo-obstruction, or celiac disease among many should be deeply studied to provide more effective treatments and clinical management. 

This special issue illustrates the cutting edge of chronic intestinal diseases treatment and the envisaged future in the management of these pathologies.

## References

[1] Gomollón F., Dignass A., Annese V., Tilg H., Van Assche G., Lindsay J.O., Peyrin-Biroulet L., Cullen G.J., Daperno M., Kucharzik T. (2017). 3rd European Evidence-based Consensus on the Diagnosis and Management of Crohn’s Disease 2016: Part 1: Diagnosis and Medical Management. J. Crohn’s Colitis.

[2] Drossman D.A., Hasler W.L. (2016). Rome IV-Functional GI Disorders: Disorders of Gut-Brain Interaction. Gastroenterology.

[3] Poon D., Law G.R., Major G., Andreyev H.J.N. (2022). A systematic review and meta-analysis on the prevalence of non-malignant, organic gastrointestinal disorders misdiagnosed as irritable bowel syndrome. Sci. Rep..

[4] Laredo V., Gargallo-Puyuelo C.J., Gomollón F. (2022). How to Choose the Biologic Therapy in a Bio-naïve Patient with Inflammatory Bowel Disease. J. Clin. Med..

[5] Mas E.B., Calvo X.C. (2022). Selecting the Best Combined Biological Therapy for Refractory Inflammatory Bowel Disease Patients. J. Clin. Med..

[6] García M.J., Rivero M., Miranda-Bautista J., Bastón-Rey I., Mesonero F., Leo-Carnerero E., Casas-Deza D., Cagigas Fernández C., Martin-Cardona A., El Hajra I. (2021). Impact of Biological Agents on Postsurgical Complications in Inflammatory Bowel Disease: A Multicentre Study of Geteccu. J. Clin. Med..

[7] Kofla-Dłubacz A., Akutko K., Krzesiek E., Jamer T., Braksator J., Grębska P., Pytrus T., Stawarski A. (2022). Selective Forms of Therapy in the Treatment of Inflammatory Bowel Diseases. J. Clin. Med..

[8] Gros M., Gros B., Mesonero J.E., Latorre E. (2021). Neurotransmitter Dysfunction in Irritable Bowel Syndrome: Emerging Approaches for Management. J. Clin. Med..

[9] Ko D., Yang H.-B., Youn J., Kim H.-Y. (2021). Clinical Outcomes of Pediatric Chronic Intestinal Pseudo-Obstruction. J. Clin. Med..

